# Lot quality assurance sampling for coverage evaluation of a new vaccine: A pilot study

**DOI:** 10.1016/j.jvacx.2024.100578

**Published:** 2024-11-01

**Authors:** Rhythm Hora, Arindam Ray, Imkongtemsu Longchar, G.R. Rio, Rashmi Mehra, Seema Singh Koshal, Amrita Kumari, Syed F. Quadri, Amanjot Kaur, Arup Deb Roy

**Affiliations:** a John Snow India, New Delhi, India; b Bill and Melinda Gates Foundation, New Delhi, India; c Directorate of Health and Family Welfare, Government of Nagaland, India

## Abstract

**Background:**

Worldwide, vaccine-preventable diseases have been a significant cause of mortality in the under-5 age group. To reduce the disease burden, new vaccines are being introduced in every country’s immunization programmes. For this to happen, high vaccination coverage is necessary. However, rapidly identifying the areas that fail to reach the expected coverage becomes cumbersome. During recent years, lot quality assurance sampling (LQAS) has been widely used in evaluating immunization coverage across the globe. The present study aims to pilot this approach for field monitoring of a new vaccine against routine concurrent field monitoring in one of the North-Eastern states of India.

**Methodology:**

For LQAS, a community-based cross-sectional study was undertaken among 55 children aged 0–23 months in all 5 Primary health centres (lots) of Medziphema block, Dimapur, Nagaland. The total sample size for LQAS was calculated based on α = 5, β = 90 using Lemeshow and Taber-LQAS table with a target level of immunization defined as 90 % and the lower limit set to 55 %. For the concurrent field monitoring, a sample of 30 children in the same age group was selected through random sampling. Pre-designed, pre-tested questionnaire for the caregivers, scripted on a digital tool was employed with verification of immunization card and caregiver’s recall. Data was analyzed using SPSS software version 25.0.

**Results:**

The study found a slight difference in the percentage of children age-appropriately vaccinated for PCV (as per the schedule) in concurrent field monitoring (93.3 %) and LQAS (90.9 %). However, no statistically significant difference was found in comparing the immunization coverage using both methodologies (p > 0.05).

**Conclusion:**

The study findings encourage that LQAS can be considered for monitoring the immunization coverage of a newly introduced vaccine. It offers the added advantage of identifying poor/low-performing pockets that require focused attention.

## Highlights

• The study highlights the importance of monitoring that follows a new vaccine introduction.
• The study stresses the increasing application of population-based surveys like LQAS for immunization coverage evaluation.
• The study compared rapid concurrent field monitoring method against LQAS method for evaluation of immunization coverage.
• The study recommends considering LQAS method for coverage evaluation of a new vaccine following its introduction.

## Introduction

Worldwide, new vaccines are being introduced in the national immunization programmes with the intent to reduce the burden of vaccine-preventable diseases (VPDs) [1], [2]. In 2021, India continued on its journey to introduce another new vaccine, namely, the Pneumococcal Conjugate Vaccine (PCV) under the ambit of the Universal Immunization Programme (UIP) that now provides vaccines against 12 VPDs to an annual cohort of close to 27 million [3], [4], [5]. However, introducing any new vaccine ushers the challenge of achieving high vaccination coverage [6].

Therefore, the successful launch of any new vaccine is often coupled with rapid concurrent field monitoring^1^ that focuses on systematic and continuous data collection and analysis to assess the implementation status of the newly introduced vaccine, thereby identifying and addressing the bottlenecks in achieving high immunization coverage [7], [8], [9].

Further, WHO recommends conducting population-based immunization coverage surveys every three to five years to evaluate the coverage of the newly introduced vaccines in the national immunization programme and compare it with the coverage of the co-administered vaccines [8]. Since PCV expansion in India happened during the pandemic and it has been more than a year of its introduction, so evaluating the coverage becomes crucial.

While the cluster sampling technique has been the most popularly used technique for evaluating immunization coverage [10], [11], of late, the lot quality assurance sampling (LQAS) technique is being successfully employed in healthcare settings [12], [13], [14], [15]. LQAS is a prompt and rapid survey method utilized to evaluate the quality of vaccination coverage following supplementary immunization activities (SIA) in pre-defined areas, such as a health district (known as “lots”), using a small sample size [16].

As LQAS is being widely used in the evaluation of immunization coverage across the globe, owing to its feasibility in rapidly identifying areas with low coverage [14], [15], [17], [18], utilizing the technique in assessing PCV coverage has been considered. Therefore, the present study aims to pilot this approach for coverage assessment of a new vaccine against routine rapid concurrent field monitoring in one of the Northeastern states (Nagaland) of India. This can be replicated in the future to rapidly assess the vaccination coverage for any other new vaccine post-introduction.

## Methodology

### Study design

A community-based cross-sectional study was undertaken in all 5 Primary health centres under the Medziphema block of Dimapur district, Nagaland (India) in May 2023. A district in Nagaland was chosen for the pilot as these areas are remote and sparsely populated areas where rapid concurrent field monitoring is non-viable. The district and block selection were done in close consultation with the Directorate of Health and Family Welfare, Government of Nagaland as these are security-compromised areas, and hence the guidance from the state is binding.

### Study participants

For the study, children aged between 0 and 23 months as of the date of the survey were considered. Only children with the availability of immunization proof (either an immunization card- Maternal & Child Protection (MCP) card or a responsible caregiver recall) and children residing in the study area for more than 6 months were included in the study. To avoid the caregivers’ recall bias for the events that occurred several months ago, verification of immunization was done by contacting the designated Auxiliary Nurse Midwife (ANMs) or Accredited Social Health Activists (ASHAs) of that area. The sample size for LQAS was calculated using Lemeshow & Taber Table [19], [20]. Setting the upper cut-off value at 90 % (Full Immunization Coverage (FIC) goal set by the Universal Immunization Programme) and the lower cut-off at 55 % (based on FIC of Nagaland reported in NFHS-5 survey) [21], [22], the sample size derived for each lot was 11 (at α = 5 %, β = 90 %). However, the decision cut-off value was considered as 8 [19], [20]. The decision rule served as a benchmark for a lot to be considered acceptable or not acceptable. If the lot had 8 or more children age-appropriately vaccinated for PCV (all doses) as per the schedule within one month of the recommended time, then immunization coverage in that lot was considered acceptable. As the study included all 5 Primary Health Centres (PHCs), each considered a distinct lot, the total sample size calculated in the LQAS was 55. Besides, the selection of 11 households in each lot was done through random sampling which included the collection of data lists of the target beneficiaries (study participants) from the ANM registers available at the centres. These data lists were then compiled to ensure random sampling before the study.

A sample of 30 children in the same age group (0–23 months) was identified. For the rapid concurrent field monitoring, 10 children from households in the catchment area across 3 of the 5 PHCs of the Medziphema block were chosen through random sampling. This is in line with the guidelines issued by the Ministry of Health and Family Welfare (MoHFW) for routine immunization (RI) monitoring which recommends visiting 10 households for house-to-house monitoring through RI/MCP card and/or verbal recall of caregiver. However, inadequate resources and time constraints resulted in the data collection only in 3 PHCs during rapid concurrent field monitoring [23].

### Study tools & data collection

**Fig. 1:**
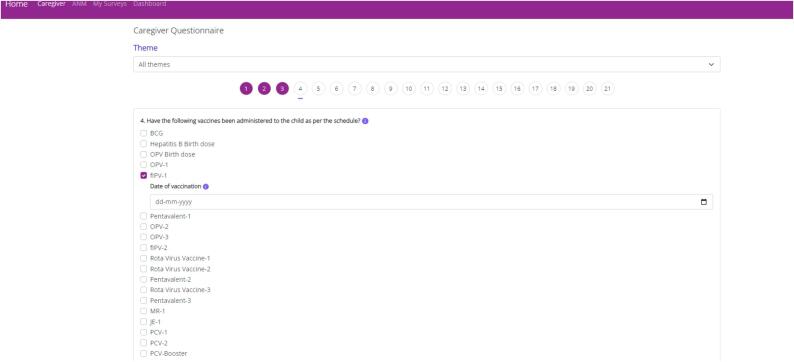
Snapshot of the digital tool.

A pre-designed, pre-tested, closed-ended English questionnaire for the caregivers, scripted on a digital tool was employed for the data collection (Fig. 1). To serve this purpose, the questionnaire was adopted and adapted from the routine immunization house-to-house monitoring format approved by the Ministry of Health and Family Welfare (MoHFW). The data was collected by verifying the immunization card and caregiver’s recall. Prior written informed consent was obtained from the caregivers for the study, and only those who agreed to contribute were included.

### Data analysis

Data was analyzed using the Statistical Package for the Social Sciences (SPSS) software version 25.0 from SPSS Inc. Descriptive statistics were used to assess the socio-demographic variables and the PCV coverage status of the sampled subjects. The chi-square test was employed as the study utilized two distinct methods to compare the PCV immunization coverage.

### Ethics approval

The study was undertaken in collaboration and consultation with the Directorate of Health and Family Welfare, Government of Nagaland. The data collected and recorded from all the participants was kept anonymous to secure their personal information.

## Results

All the study participants were residents of rural areas. Table 1 summarizes the various socio-demographic characteristics of the sampled population for both LQAS and Rapid concurrent field monitoring.

**Table 1:** Socio-demographic characteristics of study participants.

**Variable**	**LQAS**		**Rapid concurrent field monitoring**	
	**Frequency (n = 55)**	**%age**	**Frequency (n′ = 30)**	**%age**
**Age of Child**				
0–11 months	27	49.1	13	43.3
12–23 months	28	50.9	17	56.7

**Sex of child**				
Male	28	50.9	15.0	50.0
Female	27	49.1	15.0	50.0

**Primary caregiver of child**				
Mother	55	100.0	30.0	100.0

**Age of caregiver**				
<25 yrs	24	43.6	8	26.7
26–35 yrs	23	41.8	18	60.0
36–45 yrs	8	14.5	4	13.3

**Education of caregiver**				
Graduate or postgraduate	3	5.5	1	3.3
Intermediate or post-high school diploma	6	10.9	6	20.0
High school certificate	17	30.9	10	33.3
Middle school certificate	18	32.7	8	26.7
Primary school certificate	5	9.1	3	10.0
Illiterate	6	10.9	2	6.7

**Place of childbirth**				
Institutional birth	38	69.1	24	80.0
Assisted at home	9	16.4	2	6.7
Un-assisted birth	8	14.5	4	13.3

**Total**	**55**	**100.0**	**30**	**100.0**

The overall age-appropriate vaccination coverage of PCV (all doses as per the fschedule) in LQAS was 90.9 %, which was above the 90 % goal set by the UIP. Further, the age-appropriate vaccination coverage status of the PCV vaccine in all the lots was above the acceptable level. Hence, all the lots were acceptable (Table 2).

**Table 2:** Age-appropriate vaccination coverage status for PCV in LQAS.

**Lot**	**Lot sample size**	**Age-appropriate vaccination coverage for PCV**	
		**Frequency**	**%age**
Piphema	11	11	100.0 %
Pherima	11	9	81.8 %
Molvom	11	10	90.9 %
Zhuikhu	11	10	90.9 %
Ruzaphema	11	10	90.9 %

The overall age-appropriate vaccination coverage of PCV (all doses as per the schedule) in rapid concurrent field monitoring was 93.3 % (Table 3), showing that the set goal of immunization (90 %) was achieved.

**Table 3:** Age-appropriate vaccination coverage status for PCV in rapid concurrent field monitoring.

**Block**	**Sample size**	**Age-appropriate vaccination coverage for PCV**	
		**Frequency**	**%age**
Medziphema	30	28	93.3 %

**Fig. 2:**
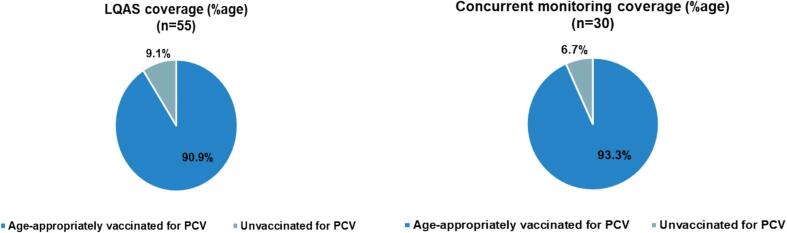
PCV coverage- LQAS vs Concurrent field monitoring.

The age-appropriate PCV vaccination coverage differed in both the methods utilized in the study. It was found to be 90.9 % in the LQAS method, whereas it was 93.3 % in the rapid concurrent field monitoring method (Fig. 2).

However, the differences in the age-appropriate vaccination coverage results of the two methodologies were not found to be statistically significant (p > 0.05) (Table 4).

**Table 4:** Chi-square test to compare rapid concurrent field monitoring and LQAS.

	Value	df	Asymp. Sig. (2-sided)	Exact Sig. (2-sided)	Exact Sig. (1-sided)
Pearson Chi-Square	0.151^a^	1	0.698	–	–
Continuity Correction^b^	0.000	1	1.000	–	–
Likelihood Ratio	0.156	1	0.693	–	–
Fisher's Exact Test	–	–	–	1.000	0.524
Linear-by-Linear Association	0.149	1	0.699	–	–
N of Valid Cases	85	–	–	–	–

All the surveyed population (n = 55 in LQAS and n′ = 30 in rapid concurrent field monitoring) had the new Maternal & Child Healthcare Card (MCP) with PCV included in it.

**Fig. 3:**
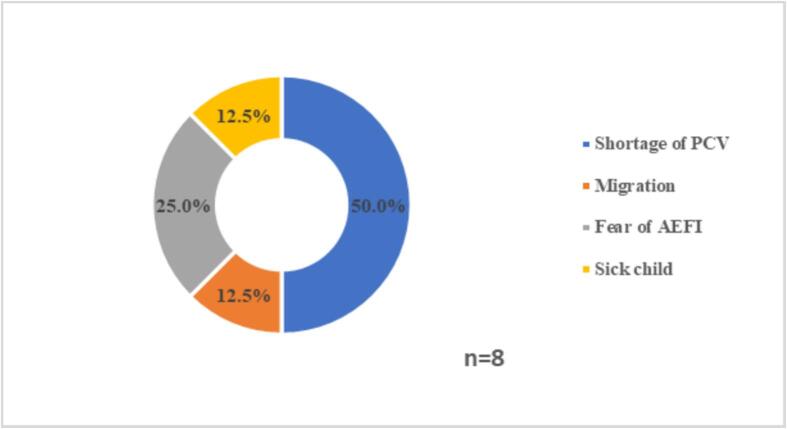
Reasons for missed PCV doses.

The caregivers of children who missed a dose of PCV were asked about the reasons for missing vaccine doses. Of the reasons cited, the shortage of PCV vaccine emerged as the primary reason for discontinuing the immunization (Fig. 3).

## Discussion

The introduction of new vaccines necessitates achieving enhanced vaccination coverage. A primary method used for evaluating the immunization coverage of newly introduced vaccines is rapid concurrent field monitoring. However, with the growing utilization of population-based surveys, such as Lot Quality Assurance Sampling (LQAS), for assessing immunization coverage [10], [12], [13], [14], this study aimed to pilot the LQAS approach in comparison with routine rapid concurrent field monitoring for the Pneumococcal Conjugate Vaccine (PCV). Punith et al. (2008) conducted a similar investigation, comparing immunization coverage using two distinct methodologies.

The findings of this study indicated that the proportion of children vaccinated on schedule for PCV was 93.3 % based on rapid concurrent field monitoring and 90.9 % using LQAS. Despite the observed difference, statistical analysis revealed no significant difference in immunization coverage between the two methods. These results align with prior studies that utilized cluster sampling and LQAS for assessing infant immunization coverage under the Universal Immunization Programme (UIP) [15].

The study further underscores the practical utility of the LQAS method in evaluating immunization coverage. Although all LQAS lots in this study demonstrated coverage exceeding the acceptable threshold, the technique can also be used to uncover areas or pockets with suboptimal coverage requiring targeted interventions.

This study has important implications for monitoring immunization coverage following the introduction of new vaccines in the future. It demonstrated that the LQAS technique could effectively evaluate the coverage of newly introduced vaccines and identify sub-regions with potential programmatic gaps. However, as this was a pilot study, the challenges of conducting this study in sparsely populated, security-compromised, remote, and hard-to-reach areas with limited resources led to a small sample size, which may have impacted the sensitivity of the decision-making process. Consequently, the necessity for a larger-scale study to assess immunization coverage in the future may be recommended. Furthermore, a larger LQAS study in larger states can be conducted to aid in identifying sub-regions/pockets that otherwise go unidentified due to the overall high coverage reported for that area. Besides, this pilot study also makes a good case to consider the LQAS technique for monitoring the immunization coverage of any newly introduced vaccine in the future.

## Conclusion

The study highlights that though there was relatively no difference in immunization coverage monitoring using both methodologies, the LQAS approach can best be utilized to determine the immunization coverage of the selected geographical region and identify problematic pockets that go unidentified due to the overall high coverage reported for that area. Additionally, conducting the LQAS study to assess immunization coverage at a larger scale can be considered.

## CRediT authorship contribution statement

**Rhythm Hora:** Writing – original draft, Visualization, Software, Methodology. **Arindam Ray:** Writing – review & editing. **Imkongtemsu Longchar:** Project administration, Conceptualization. **G.R. Rio:** Supervision. **Rashmi Mehra:** Validation, Methodology. **Seema Singh Koshal:** Supervision, Investigation, Data curation. **Amrita Kumari:** Supervision, Conceptualization. **Syed F. Quadri:** Visualization, Supervision, Conceptualization. **Amanjot Kaur:** Resources, Investigation, Formal analysis. **Arup Deb Roy:** Writing – review & editing, Supervision, Project administration, Conceptualization.

## Declaration of competing interest

The authors declare that they have no known competing financial interests or personal relationships that could have appeared to influence the work reported in this paper.

## Data Availability

Data will be made available on request.

## Footnotes

1 **Rapid concurrent field monitoring** focuses on the systematic and continuous collection and analysis of data for measuring the process & progress of the programme. The main objective is to track whether actual results are being achieved as planned. Standardized data collection formats and operating procedures have been developed by MoHFW to monitor the provision of routine immunization services for all antigens offered through UIP to detect coverage gaps. It is done at the immunization session site and house-to-house (community-led) using the standard monitoring formats. For rapid concurrent field monitoring, a pool of monitors from the government departments and development partners are identified and trained on the immunization schedule, the process, and the information to be collected. The field visits are planned as per the micro plan, considering three “Ws”: where to conduct visits (priority areas); when to conduct visits (on immunization session days after informing the health workers); and what to do during visits (review data and previous supervision and monitoring reports).
