# Supplementary material for: Lot quality assurance sampling for coverage evaluation of a new vaccine: A pilot study
Source: Vaccine X. 2024 Nov 1;21:100578. doi: 10.1016/j.jvacx.2024.100578 (PMC11570966; doi:10.1016/j.jvacx.2024.100578)
Supplement: Supplementary Data 1 [file mmc1.doc]

**. Immunization - House to House Monitoring Format**

**Encircle appropriate options. For (*) marked questions multiple responses are allowed.**

| Date: _ _ / _ _ / _ _ Monitoring time: _ _ : _ _ to _ _ : _ _ Type of Monitoring: RI / LQAS; ………………………. State/UT: ………………………………………..; District: ………………………………………………  Village/Mohalla/Ward…………………………..……………………; Area setting: Rural / Peri-urban / Urban; If Rural or Peri-Urban area: Sub center (R): ……………………………………………………………………………...  Block / Urban name: ………………………………… ; If Urban: NUHM City: Yes / No; Name of ANM: ………….………………………..Planning Unit name (Rural or Urban): ……………………………………………………… | | | | | | | | | | | | | | | | | | | | | | | | | | | | | | | | |
| --- | --- | --- | --- | --- | --- | --- | --- | --- | --- | --- | --- | --- | --- | --- | --- | --- | --- | --- | --- | --- | --- | --- | --- | --- | --- | --- | --- | --- | --- | --- | --- | --- |
| HRA (as in RI Micro-plan): Yes / No  (select migratory or non-migratory) | | *Migratory area | | **a.** Normal accessible area: **b.** Hard to reach area **c.** Vacant sub center area (unserved area) **d.** Urban slum **e.** Peri-urban area **f.** Area with Nomads **g.** Brick Kiln **h.** Construction site **i.**  Industrial compound **j.** Area with vaccine hesitancy **k.** Area with underserved population  **l.** Area with VPD Outbreak **m.** Others – Riverine area, Fishermen colony, Forest area, Tea Garden area etc. | | | | | | | | | | | | | | | | | | | | | | | | | | | | |
|  |  | *Non-migratory | |  |  |  |  |  |  |  |  |  |  |  |  |  |  |  |  |  |  |  |  |  |  |  |  |  |  |  |  |  |
| Name of Monitor-1: ………………………………………………………; Organization: JSI; Designation: …………………………….  Name of Monitor-2: ………………………………………………………; Organization: JSI; Designation: ……………………………. | | | | | | | | | | | | | | | | | | | | | | | | | | | | | | | | |
| **Particulars of the child and age specific vaccination status.** | | | **House-1** | | | | **House-2** | | | **House-3** | | | **House-4** | | | **House-5** | | | **House-6** | | | **House-7** | | | **House-8** | | | **House-9** | | | **House-10** | |
| 1 | Name of the selected child **(0-23 months)** | |  | | | |  | | |  | | |  | | |  | | |  | | |  | | |  | | |  | | |  | |
| 2 | Age in completed months (0-23 months) | |  | | | |  | | |  | | |  | | |  | | |  | | |  | | |  | | |  | | |  | |
| 3 | Who is the main source of information on the selected child?  [Mother = M; Father = F, Grandparents = G , O = Other] | | M / F / G / O | | | | M / F / G / O | | | M / F / G / O | | | M / F / G / O | | | M / F / G / O | | | M / F / G / O | | | M / F / G / O | | | M / F / G / O | | | M / F / G / O | | | M / F / G / O | |
| 4 | Name of the Father/Mother of the child | |  | | | |  | | |  | | |  | | |  | | |  | | |  | | |  | | |  | | |  | |
| 5 | Age of caregiver (in years) | |  | | | |  | | |  | | |  | | |  | | |  | | |  | | |  | | |  | | |  | |
| 6 | Religion (**H**=Hindu / **M**=Muslim / **O**=Others) | | H / M / O | | | | H / M / O | | | H / M / O | | | H / M / O | | | H / M / O | | | H / M / O | | | H / M / O | | | H / M / O | | | H / M / O | | | H / M / O | |
| 7 | Caste (Gen, OBC, SC/ST): | | Gen/ OBC/ SC/ST | | | | Gen/ OBC/ SC/ST | | | Gen/ OBC/ SC/ST | | | Gen/ OBC/ SC/ST | | | Gen/ OBC/ SC/ST | | | Gen/ OBC/ SC/ST | | | Gen/ OBC/ SC/ST | | | Gen/ OBC/ SC/ST | | | Gen/ OBC/ SC/ST | | | Gen/ OBC/ SC/ST | |
| 8 | Sex of the selected child: **M**=Male / **F**=Female | | M / F | | | | M / F | | | M / F | | | M / F | | | M / F | | | M / F | | | M / F | | | M / F | | | M / F | | | M / F | |
| 9 | Place of delivery: **G)** Govt – Hospital, **P)** Private Hospital, **H)** Home | |  | | | |  | | |  | | |  | | |  | | |  | | |  | | |  | | |  | | |  | |
| 10 | Date of birth (In dd/mm/yy). [if not known, write NA] | | G / P / H | | | | G / P / H | | | G / P / H | | | G / P / H | | | G / P / H | | | G / P / H | | | G / P / H | | | G / P / H | | | G / P / H | | | G / P / H | |
| 11 | Education level of caregiver (Graduate and Above, Higher Secondary/Senior Secondary, Matric/Secondary, Middle, Primary, Graduate and Above) | | Graduate and Above, Higher Secondary/Senior Secondary, Matric/Secondary, Middle, Primary, Graduate and Above | | | | Graduate and Above, Higher Secondary/Senior Secondary, Matric/Secondary, Middle, Primary, Graduate and Above | | | Graduate and Above, Higher Secondary/Senior Secondary, Matric/Secondary, Middle, Primary, Graduate and Above | | | Graduate and Above, Higher Secondary/Senior Secondary, Matric/Secondary, Middle, Primary, Graduate and Above | | | Graduate and Above, Higher Secondary/Senior Secondary, Matric/Secondary, Middle, Primary, Graduate and Above | | | Graduate and Above, Higher Secondary/Senior Secondary, Matric/Secondary, Middle, Primary, Graduate and Above | | | Graduate and Above, Higher Secondary/Senior Secondary, Matric/Secondary, Middle, Primary, Graduate and Above | | | Graduate and Above, Higher Secondary/Senior Secondary, Matric/Secondary, Middle, Primary, Graduate and Above | | | Graduate and Above, Higher Secondary/Senior Secondary, Matric/Secondary, Middle, Primary, Graduate and Above | | | Graduate and Above, Higher Secondary/Senior Secondary, Matric/Secondary, Middle, Primary, Graduate and Above | |
| 12 | Employment status of caregiver (employed/ unemployed) | | employed/ unemployed | | | | employed/ unemployed | | | employed/ unemployed | | | employed/ unemployed | | | employed/ unemployed | | | employed/ unemployed | | | employed/ unemployed | | | employed/ unemployed | | | employed/ unemployed | | | employed/ unemployed | |
| Interact with caregiver and refer ready reckoner to ascertain vaccination status. If MCP/RI card is available, monitor to write date (dd/mm/yy) for vaccines received and “No” for missed vaccines. If card / legible and/or logical date is not available, ascertain on verbal recall, mention “Yes” for vaccine received / “No” for missed vaccine. Mention “NA” for vaccine not due for age in RI / not introduced in the district/state. | | | | | | | | | | | | | | | | | | | | | | | | | | | | | | | | |
| 13 | **Hep B Birth dose** | |  | | |  | | |  | | |  | | |  | | |  | | |  | | |  | | |  | | |  | | |
|  | **bOPV-0 dose** | |  | | |  | | |  | | |  | | |  | | |  | | |  | | |  | | |  | | |  | | |
|  | BCG | |  | | |  | | |  | | |  | | |  | | |  | | |  | | |  | | |  | | |  | | |
|  | OPV-1 | |  | | |  | | |  | | |  | | |  | | |  | | |  | | |  | | |  | | |  | | |
|  | Rotavirus-1 | |  | | |  | | |  | | |  | | |  | | |  | | |  | | |  | | |  | | |  | | |
|  | fIPV-1 | |  | | |  | | |  | | |  | | |  | | |  | | |  | | |  | | |  | | |  | | |
|  | PCV 1 | |  | | |  | | |  | | |  | | |  | | |  | | |  | | |  | | |  | | |  | | |
|  | Pentavalent-1 | |  | | |  | | |  | | |  | | |  | | |  | | |  | | |  | | |  | | |  | | |
|  | OPV-2 | |  | | |  | | |  | | |  | | |  | | |  | | |  | | |  | | |  | | |  | | |
|  | Rotavirus- 2 | |  | | |  | | |  | | |  | | |  | | |  | | |  | | |  | | |  | | |  | | |
|  | Pentavalent-2 | |  | | |  | | |  | | |  | | |  | | |  | | |  | | |  | | |  | | |  | | |
|  | OPV-3 | |  | | |  | | |  | | |  | | |  | | |  | | |  | | |  | | |  | | |  | | |
|  | Rotavirus-3 | |  | | |  | | |  | | |  | | |  | | |  | | |  | | |  | | |  | | |  | | |
|  | fIPV 2 | |  | | |  | | |  | | |  | | |  | | |  | | |  | | |  | | |  | | |  | | |
|  | PCV2 | |  | | |  | | |  | | |  | | |  | | |  | | |  | | |  | | |  | | |  | | |
|  | Pentavalent-3 | |  | | |  | | |  | | |  | | |  | | |  | | |  | | |  | | |  | | |  | | |
|  | **Vaccination details & reasons for missed dose(s) as applicable** | | **House-1** | | | **House-2** | | | **House-3** | | | **House-4** | | | **House-5** | | | **House-6** | | | **House-7** | | | **House-8** | | | **House-9** | | | **House-10** | | |
|  | MR-1 | |  | | |  | | |  | | |  | | |  | | |  | | |  | | |  | | |  | | |  | | |
|  | PCV Booster | |  | | |  | | |  | | |  | | |  | | |  | | |  | | |  | | |  | | |  | | |
|  | JE-1 (where applicable) | |  | | |  | | |  | | |  | | |  | | |  | | |  | | |  | | |  | | |  | | |
| 14 | If received all due doses, did the child complete age specific vaccination as per UIP schedule? (Don't consider Hepatitis-B birth dose & bOPV-0) | | Y / N / NA | | | | Y / N / NA | | | Y / N / NA | | | Y / N / NA | | | Y / N / NA | | | Y / N / NA | | | Y / N / NA | | | Y / N / NA | | | Y / N / NA | | | Y / N / NA | |
| 15 | If child has missed any due vaccine dose(s), ascertain reason(s) from caregiver (you can list upto 4 reasons) | | __ | | __ | | __ | __ | | __ | __ | | __ | __ | | __ | __ | | __ | __ | | __ | __ | | __ | __ | | __ | __ | | __ | __ |
|  |  |  | __ | | __ | | __ | __ | | __ | __ | | __ | __ | | __ | __ | | __ | __ | | __ | __ | | __ | __ | | __ | __ | | __ | __ |
| 16 | Was the RI Card updated? (Y/N) | | Y/N | | | | Y/N | | | Y/N | | | Y/N | | | Y/N | | | Y/N | | | Y/N | | | Y/N | | | Y/N | | | Y/N | |
